# mTOR in Viral Hepatitis and Hepatocellular Carcinoma: Function and Treatment

**DOI:** 10.1155/2014/735672

**Published:** 2014-04-02

**Authors:** Zhuo Wang, Wei Jin, Hongchuan Jin, Xian Wang

**Affiliations:** Department of Medical Oncology, Institute of Clinical Science, Sir Runrun Shaw Hospital, School of Medicine, Zhejiang University, Hangzhou, China

## Abstract

As the fifth most common cancer in men and the eighth most common cancer in women, hepatocellular carcinoma (HCC) is the leading cause of cancer-related deaths worldwide, with standard chemotherapy and radiation being minimally effective in prolonging survival. Virus hepatitis, particularly HBV and HCV infection is the most prominent risk factor for HCC development. Mammalian target of rapamycin (mTOR) pathway is activated in viral hepatitis and HCC. mTOR inhibitors have been tested successfully in clinical trials for their antineoplastic potency and well tolerability. Treatment with mTOR inhibitor alone or in combination with cytotoxic drugs or targeted therapy drug scan significantly reduces HCC growth and improves clinical outcome, indicating that mTOR inhibition is a promising strategy for the clinical management of HCC.

## 1. Introduction

Hepatocellular carcinoma (HCC) is a malignant tumor whose incidence is increasing in many countries. It is the fifth most common cancer in men and the eighth most common cancer in women. HCC is the leading cause of cancer-related deaths worldwide, with standard chemotherapy being minimally effective in prolonging survival [1].

Among many factors such as environmental pollution, fatty liver, and excessive alcohol consumption, virus hepatitis, particularly HBV and HCV infection, has been considered as the most important high risk factor of HCC, especially in Asian countries. At the molecular level, mammalian target of rapamycin (mTOR) pathway was found to be associated with HCC development including chronic viral hepatitis [2, 3]. Inhibitors of mTOR were thus postulated to be prominent for the clinical treatment of HCC.

## 2. mTOR

### 2.1. Structure of mTOR Complex

mTOR is a member of PI3K-related protein kinases (PIKK). The structure of mTOR is similar to other PIKK family members. The amino terminus of mTOR is a cluster of HEAT (Huntingtin, Elongation factor 3, A subunit of protein phosphatase 2A, and TOR1) repeats, followed by FAT (FRAP, ATM, and TRRAP) domain, FKBP12-rapamycin binding (FRB) domain, Ser/Thr kinase catalytic domain, and the carboxyl-terminal FAT (FATC) domain. HEAT domain can mediate protein-protein interactions and FRB domain is a conserved 11 kDa region necessary for the binding of rapamycin and regulatory-associated protein of mTOR (RAPTOR) [4].

**Figure 1:**
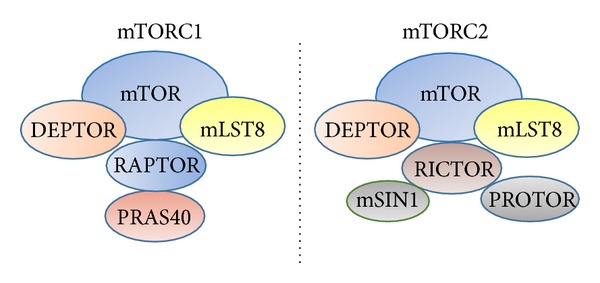
The structure of mTORC1 and mTORC2. The core mTOR machinery consists of mTOR, DEPTOR, and mLST8. The combination of core mTOR machinery with different proteins constitutes mTOR1 and mTORC2.

According to different subunits, mTOR can be formed as two kinds of complexes, mTORC1 and mTORC2 (Figure 1). Both mTOR complexes contain mTOR, DEP domain-containing mTOR-interacting protein (DEPTOR), and mammalian lethal with SEC13 protein 8 (mLST8). The unique components of mTORC1 are regulatory-associated protein of mTOR (RAPTOR) and proline-rich Akt substrate of 40 Kda (PRAS40). mTORC2 possesses rapamycin-insensitive companion of mTOR (RICTOR), protein observed with RICTOR (PROTOR), and mammalian stress-activated map kinase-interacting protein 1 (mSIN1). Among them, PRAS40 is a negative regulator of mTOR and has a conserved leucine charge domain (LCD) which can be phosphorylated by AKT [5, 6]. mLST8 can mediate protein-protein interactions while mSIN1 contains a Ras-binding domain (RBD) and a pleckstrin homology which can interact with phospholipid. Currently, the structures of RICTOR and PROTOR are still not clear.

Rapamycin can inhibit the mTORC1 but not mTORC2, because rapamycin binds with FKBP12 to disrupt the interaction of mTOR with RAPTOR but not RICTOR [7–9]. The rapamycin-induced dissociation of mTOR from RAPTOR eventually prevents interaction of the mTOR with a number of substrates [10, 11]. However, long-term rapamycin treatment can inhibit mTORC2 [12]. This effect may involve the changes of intracellular pool of mTOR and thus reduce the assembly of mTORC2.

### 2.2. Regulation of mTOR Activation

**Figure 2:**
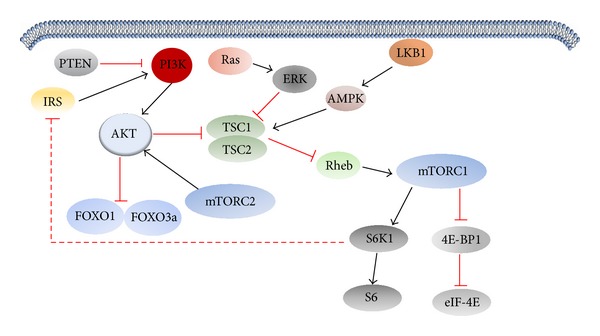
The regulation of mTOR. The activity of mTOR can be regulated by PI3K-Akt and LKB1-AMPK pathway. Activated mTOR regulates transcriptional activity of FOXO1-FOXO3a and protein translation by pS6 and eIF-4E.

mTORC1 can be activated by diverse factors, such as growth factors, various cytokines, Toll-like receptor ligands, cell energy levels, hypoxia, and DNA damage. The activation of mTORC1 plays an important role in protein synthesis, ribosome biogenesis, and autophagy. Activated mTORC1 can phosphorylate the downstream signaling molecules including S6K1 or RPS6KN1 (ribosomal protein S6 kinase, 70 kDa, polypeptide 1) and eukaryotic translation initiation factor-binding protein 1 (4E-BP1). Activation of S6K1 can promote the expression of ribosomal protein and translation regulating protein to regulate protein syntheses. Nonphosphorylated 4E-BP1 can bind to eIF-4E to inhibit mRNA translation. Once phosphorylated by active mTOR, 4E-BP1 are dissociated from eIF-4E so that eIF-4E can bind to other translation initiation factors to initiate protein translation [13, 14]. Tuberous sclerosis complex 1- (TSC1-) TSC2 tumor suppressor complex is a negative regulator of mTOR. As a GTP activating protein (GAP), TSC2 or tuberin inactivates Ras homologue enriched in brain (Rheb) which can directly bind to and activate mTOR. TSC1 or hamartin does not have a GAP domain but it acts as a stabilizer of TSC2 by preventing it from degradation. The activity of TSC1-TSC2 is regulated by protein phosphorylation. Activated PI3K-Akt signaling can phosphorylate and inhibit TSC1-TSC2 while LKB1-AMPK can activate TSC1-TSC2 by phosphorylation at different residues (Figure 2) [15, 16].

The activation of mTORC1 can be regulated by several factors through signaling pathways including PI3K/Akt/mTOR, LKB1/AMPK/mTOR, and MAPK pathway. Once activated by extracellular signals such as growth factors and nutrient, PI3K can phosphorylate PIP2 to form PIP3 [17]. As a result, Akt and its activator phosphoinositide-dependent protein kinases 1 (PDK1) translocate to the plasma membrane by binding to PIP3. When phosphorylated at Thr308 and Ser473, Akt can activate mTOR by inhibiting TSC1-TSC2 [18]. In addition, Akt can suppress PRAS40 by phosphorylation to eliminate its inhibition on mTORC1.

Studies have shown that acids metabolism in mammalian cells is adjusted by LKB1/AMPK/mTOR signal pathway. mTOR pathway can be activated by adenosine monophosphate-activated protein kinases (AMPKs) that in turn can be activated by LKB1. AMPKs change their conformation in response to the intracellular AMP/ATP ratio. When AMP/ATP ratio drops, LKB1 can bind to AMPK subunit to activate AMPK by phosphorylation. The activation of AMPK will inhibit mTOR activation, reduce protein synthesis, and inhibit cell proliferation [19].

In addition, mTOR activity can be regulated in response to the availability of amino acids [20, 21]. Ras-related (Rag) GTPases, a family of four related small GTPase, are responsible for amino acid-regulated mTOR activity [22, 23]. Rag GTPases interact with the RAPTOR, the subunit of mTORC1, in an amino acid-dependent manner, allowing interaction of mTORC1 with Rheb. The Rags exist as obligate heterodimers. Depletion of either heterodimer partners can inhibit mTORC1 while overexpression of the heterodimer will rescue mTORC1 from suppression by amino acid withdrawal. Some specific amino acids may play distinct roles in the regulation of mTOR activity. For example, glutamine in combination with leucine activates mammalian TORC1 (mTORC1) by enhancing glutaminolysis and *α*-ketoglutarate production [24]. Inhibition of glutaminolysis prevented activation of RagB and mTORC1 while constitutively active Rag heterodimer activated mTORC1 in the absence of glutaminolysis. Conversely, enhanced glutaminolysis or a cell-permeable *α*-ketoglutarate analog stimulated lysosomal translocation and activation of mTORC1.

## 3. mTOR and Viral Hepatitis

### 3.1. mTOR and HBV Infection

Chronic HBV infection is a crucial factor for the development of HCC. In HepG2 cells, HBV RNA transcription and subsequent DNA replication were inhibited by the expression of a constitutively active Akt1 [25]. This inhibition of HBV gene transcription seems to be mediated by mTOR activation since rapamycin can abolish this inhibition. Furthermore, inhibitors of PI3K, Akt, and mTOR can increase the transcription of viral RNA as well as the replication of HBV DNA in HBV-overexpressing cells. Consistently, expression of HBsAg was much higher in adjacent tissues than in tumor tissues that contain high level of PI3K-Akt activity [26].

In contrast to wild type pre-S antigen, HBV pre-S mutants are viral oncoproteins to induce endoplasmic reticulum (ER) stress in ground glass hepatocytes (GGHs) that have been recognized as the precursor lesions of HCC [27]. In addition, the existence of pre-S mutants in serum of HBV carriers can predict the development of HCC. Interestingly, the expression of pre-S mutants is associated with the activation of Akt/mTOR signaling in HCC cells [28]. Pre-S mutants may upregulate VEGFR-2 to activate Akt/mTOR which can be attenuated by VEGF-A neutralization.

Intriguingly, the activation of mTOR can repress HBsAg synthesis by facilitating the interaction of histone deacetylase 1 (HDAC1) with Yin Yang1 (YY1), a transcription factor which bound to pre-S1 promoter [29]. Such a feedback regulation of HBsAg expression during HBV-initiated tumorigenesis is that mTOR inhibitors may activate HBV replication in patients with chronic HBV infection. Indeed, everolimus was associated with the risk of  HBV reactivation [30]. In addition, the knockdown of histone deacetylase 1 (HDAC1) can abolish this inhibitory effect of the mTOR on pre-S transcription. The HDAC1 inhibitors that have been intensively under evaluation for their anticancer effect may also lead to the reactivation of HBV.

HBV X (HBx) protein encoded by the HBV X gene plays a crucial role in the pathogenesis of HBV-related HCC by promoting cell cycle progression, inactivates negative growth regulators, and binds to and inhibits the expression of p53 tumor suppressor gene and other tumor suppressor genes and senescence-related factors [31]. The expression of mTOR and PI3K/Akt in HCC cells can be increased by HBx transfection [32]. Interestingly, HBx transfection increased the formation of autophagosomes and autolysosomes and upregulated microtubule-associated protein light chain 3, beclin 1, and lysosome-associated membrane protein 2a. HBx-induced autophagy was further increased by mTOR inhibitor rapamycin but blocked by treatment with the PI3K-Akt inhibitor LY294002, indicating that HBx activates autophagy through PI3K-Akt-mTOR pathway. HBx activated mTOR seems to depend on I*κ*B kinase *β* (IKK*β*) [33]. IKK*β* inhibitor Bay 11-7082 or silencing IKK*β* expression using siRNA reversed HBx-induced S6K1 activation, HBx upregulated cell proliferation, and vascular endothelial growth factor (VEGF) production. Similarly, mTOR inhibition reduced the growth, invasion, epithelial-to-mesenchymal transition, and metastasis of HBx-expressing HCC cells [34]. In the HBx transgenic mouse model, pIKK*β*, pS6K1, and VEGF expressions were higher in cancerous than noncancerous liver tissues. Furthermore, pIKK*β* levels were strongly correlated with pTSC1 and pS6K1 levels in HBV-associated human hepatoma tissues, and higher pIKK*β*, pTSC1, and pS6K1 levels were correlated with a poor prognosis in these patients.

### 3.2. mTOR and HCV Infection

**Figure 3:**
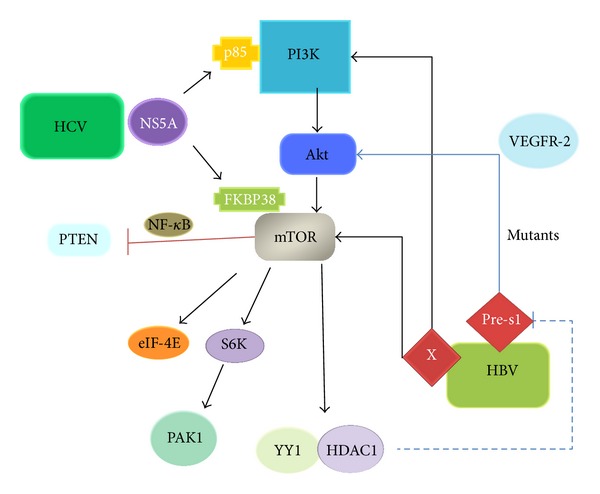
Regulation and function of mTOR in viral hepatitis and HCC development. In HCV infection, NS5A can activate mTOR through PI3K/Akt pathway or impair the combination between mTOR and FKBP38. The complex formed by mTOR and NF-*κ*B can downregulate the expression of PTEN. In HBV infection, pre-S1 can activate Akt/mTOR pathway through upregulation of VEGFR-2. YY1-HDAC1 complex can inhibit the transcription from the pre-S1 promoter as a negative feedback. HBx can increase the expression of mTOR and PI3K/Akt. S6K can activate PAK1 to regulate actin cytoskeleton and cell motility.

HCV infection contributes to the rising incidence of HCC in many developed countries, such as Spain, France, Italy, and Japan, where the proportion caused by the HCV ranges from 50% to 70% [35]. HCV nonstructural protein NS5A is a crucial factor in viral replication and diverse cellular events. NS5A can activate PI3K-mTOR signaling by directly binding to the p85 subunit of PI3K (Figure 3) [36]. Inhibition of PI3K abrogated NS5A-activated mTOR. In addition, NS5A can interact with FKBP38, an immunosuppressant FK506-binding protein. NS5A activated mTOR by releasing it from FKBP38 even in the absence of serum [37]. Rapamycin or NS5A knockdown can block S61 K and 4E-BP1 phosphorylation that were increased in HCV replicon cells and NS5A-Huh7 cell.

Strikingly, HCV infection can activate mTOR in an autophagy-dependent manner. HCV induced autophagy by upregulating beclin 1 to activate mTOR signaling pathway, which in turn promoted hepatocyte growth [38]. HCV induced mTOR activated and phosphorylation of eIF-4E were impaired in autophagy-deficient HCC cells.

Stable NS5A expression in HCC cells led to the resistance to apoptosis that was abolished by the silence of FKBP38 through RNA interference [39]. Moreover, NS5A can repress the activation of caspase-3 and poly(ADP-ribose) polymerase which was abrogated by rapamycin or NS5A knockdown, indicating that NS5A suppresses apoptosis specifically through mTOR pathway.

Interestingly, mTOR can influence the regulation of HCV RNA replication [40]. For example, mTOR downstream kinase p21-activated kinase 1 (PAK1) has been found to take part in antiviral signaling. The suppression of PAK1 by PI3K inhibitor or rapamycin enhanced viral RNA and protein affluence in established replicon HCV cell lines. Similarly, knockdown of S6K abolished PAK1 phosphorylation and enhanced HCV RNA affluence while knockdown of eIF-4E increased viral RNA affluence without affecting PAK1 phosphorylation.

## 4. mTOR and HCC

As an important part of PI3K/Akt pathway which is critical to cancer development, mTOR was aberrantly activated in most if not all human carcinogenesis. mTOR was activated in precancerous cirrhotic tissues in addition to chronic viral hepatitis tissues [2]. A small-scale immunohistochemistry staining analysis revealed that 33 out of 73 (45%) HCC patients had increased expression of total S6k which correlated with mTOR activation as well as tumor nuclear grade and tumor size [41]. Moreover, in a larger cohort of HCC patients, mTOR pathway was more remarkably altered in tumors with poor differentiation, high TNM stage, vascular invasion, and other poor prognostic features [42]. The expression of pS6 was further confirmed as an independent prognostic factor for HCC.

The activation of mTOR can confer many growth advantages to cancer stem or progenitor cells such as promoting cell proliferation and resistance to apoptosis induced by various stress signals such as hypoxia and nutrient deficiency. In addition, mTOR can regulate telomerase activity in hepatocarcinogenesis. Treating HCC cells (SMMC-7721) with rapamycin significantly reduced telomerase activity by downregulating hTERT protein level but not hTERT transcription, indicating [43].

Neovascularization in tumor is closely associated with tumor growth. Tumors that form as a result of mTOR activation are highly vascularized and inhibition of mTOR by rapamycin can diminish the process of angiogenesis. The activation of mTORC1 can promote a variety of angiogenesis-related proteins, such as hypoxia-inducible factor *α* (HIF*α*) and vascular endothelial growth factor (VEGF). Under hypoxia condition, cancer cells can produce HIF*α* through mTOR-dependent manner [43]. When oxygen is sufficient, activated mTOR can promote the translation of HIF*α* mRNA by the activation of 4E-BP1 or S6K1 [44]. Inhibition of mTOR activity in human hepatoma cells reduced HIF*α* expression without reducing its mRNA or promoting its degradation [45]. In addition to affecting its expression, mTOR can also directly regulate the activity of HIF*α* [46]. Activation of mTOR by Rheb overexpression potently enhances the activity of HIF*α* and VEGF secretion during hypoxia. RAPTOR directly interacts with HIF*α* which requires an mTOR signaling (TOS) motif located in the N terminus of HIF*α*. The mutant of HIF*α* lacking this TOS motif was unable to bind to the coactivator CBP/p300 and lost its transcriptional activity and proangiogenesis function.

Autophagy plays a crucial role in tumor suppression by eliminating damage cells. mTOR has a regulatory role for autophagy and malignant cells often exhibit defects in autophagy [47]. Autophagy-deficient mice such as beclin 1 heterozygous mice have an increased incidence of spontaneous tumors and also can accelerate the development of precancerous lesions induced by hepatitis B infection [48]. Therefore, mTOR may indirectly induce tumorigenesis by the suppression of autophagy.

4E-BP1 is a downstream mTORC1 key factor in regulating cell proliferation. Activated eIF4E preferably promote translation of tumor-related genes such as cell cycle regulatory proteins or antiapoptotic proteins like MCL1 [49]. While the relevance of mTORC1 to human carcinogenesis has been well-documented, whether mTORC2 is critical to human cancer development remains unknown. Recently, RICTOR, the unique structure of mTORC2, was found to be necessary to the tumor formation of PTEN-deficient prostate epithelial cells in nude mice, indicating that mTORC2 can function in synergy with PI3K to promote tumorigenesis [50]. In addition, inhibition of mTORC2 reduced proliferation and anchorage-independent growth of human cancer cells by inducing downregulation of cyclin D1 and cell cycle arrest at G1 phase [51].

Hepatic steatosis is a risk factor for HCC in addition to chronic viral hepatitis. PTEN expression is downregulated in the livers of rats and humans having steatosis, which was accompanied by high plasma levels of fatty acids [52]. Unsaturated fatty acids can downregulate PTEN expression via activation of a complex formed by mTOR and NF-kB in HepG2 cells.

Aberrant lipogenesis plays a pivotal role in the development of human HCC [53]. The AKT-mTORC1-S6K1 pathway facilitated lipogenesis via posttranscriptional and transcriptional mechanisms. In a NASH liver, activation of AKT and the mTOR pathway in turn triggered the development of HCC [54]. mTORC1 is crucial for the activation of the sterol regulatory element-binding proteins (SREBPs), primary transcription factors regulating genes involved in lipid and sterol synthesis [55, 56]. For example, fatty acid synthase (FAS) is encoded by a target gene of SREBP and is upregulated in some human cancers [57]. Overexpression of FAS is an early phenomenon in chemically, spontaneous, and hormonally induced rat hepatocarcinogenesis that was found to be associated with the activation of PI3K-Akt pathway [58, 59]. In addition, mTOR pathway can upregulate glycolysis in HCC [60]. It is crucial to the transcriptional regulation of glucose transporters and various rate-limiting glycolytic enzymes such as pyruvate kinase M2 (PKM2) [61]. The levels of PKM2 expression are upregulated in human cancer cells to stimulate the transactivation of glycolytic genes [62–65]. Transcription factors including HIF*α* and c-Myc are important to such transactivation effect of mTOR signaling [66, 67].

## 5. mTOR Inhibitors for HCC Treatment

mTOR inhibitors inhibit mTOR complex 1 (mTORC1) mainly through interacting with FK506-binding protein 12 (FKBP12). At present, there are 3 analogues of rapamycin with potent biological properties and pharmacokinetics have been tested in clinical trials, RAD001 (everolimus), CCI-779 (temsirolimus), and AP23573 (deforolimus). RAD001 (everolimus) is an orally bioavailable analogue and CCI-779 (temsirolimus) is a soluble ester analogue while AP23573 (deforolimus) is a nonprodrug analogue of rapamycin. mTOR inhibitors have been tested successfully in clinical trials for their antineoplastic potency and well tolerability in different malignancies, including renal cell carcinoma, pancreatic neuroendocrine tumors, subependymal giant cell astrocytomas, and breast cancer [68–72].

mTOR inhibition significantly reduces HCC growth and improves survival primarily via antiangiogenesis. After the treatment of mTOR inhibitor sirolimus for 4 weeks, rats implanted with hepatoma cells had significantly longer survival and developed smaller tumors, fewer extrahepatic metastases, and less ascites than controls [73]. Sirolimus treatment reduced intratumor microvessel density, leading to extensive necrosis. Moreover, vascular sprouting and tube formation of aortic rings were also impaired.

Basically, mTOR inhibitors are well tolerated. To evaluate the best dosing schedules, thirty-nine patients with locally advanced or metastatic HCC (Child-Pugh class A or B) were enrolled in an open-label phase 1 study and randomly assigned to daily (2.5–10 mg) or weekly (20–70 mg) everolimus in a standard 3 + 3 dose-escalation design. Dose-limiting toxicities (DLTs) occurred in five of 21 patients in the daily and two of 19 patients in the weekly cohort. Daily and weekly maximum tolerated doses (MTDs) were 7.5 mg and 70 mg, respectively. Grade 3/4 adverse events with a ≥ 10% incidence were thrombocytopenia, hypophosphatemia, and alanine transaminase (ALT) elevation. In four hepatitis B surface antigen- (HBsAg-) seropositive patients, grade 3/4 ALT elevations were accompanied by significant increases in serum HBV levels. The incidence of hepatitis flare in HBsAg-seropositive patients with and without detectable serum HBV DNA before treatment was 46.2% and 7.1%, respectively.

Another phase 1 trial of temsirolimus combined with sorafenib showed that the maximum-tolerated dose (MTD) was temsirolimus 10 mg weekly plus sorafenib 200 mg twice daily [74]. Grade 3 or 4 adverse events were hypophosphatemia, infection, thrombocytopenia, hand-foot skin reaction (HFSR), and fatigue. There is another trial that showed that the MTD of everolimus in combination with standard-dose sorafenib was 2.5 mg daily [75]. Most common adverse events are diarrhea, HFSR, and thrombocytopenia.

The randomised clinical trial to compare two everolimus dosing schedules showed 7.5 mg better than 70 mg daily in patients with HCC. Disease control rates in the daily and weekly cohorts were 71.4% and 44.4%, respectively [76]. Treatment of HCC patients with mTOR inhibitors can induce temporary PR (partial response) or SD (stable disease) [77]. In 21 advanced HCC patients treated with sirolimus once daily, one had PR and five had SD at 3 months. The median survival was 6.5 months (0.2–36 months).

Interestingly, the intraliver and intra-abdominal growths of patient-derived hepatocellular carcinoma xenografts were inhibited by bevacizumab plus rapamycin treatment to a significantly greater degree than bevacizumab or rapamycin monotherapy [78]. Reductions in tumor growth by bevacizumab plus rapamycin were associated with reductions in tumor microvessel density as well as the expression of VEGF, cyclin D1, and cyclin B1. Eventually, mouse survival was greatly prolonged after the combination treatment.

In addition, mTOR inhibitor can suppress tumor growth and sensitize tumor cell to chemotherapy or other target therapy [79–82]. For example, RAD001 alone can repress cell proliferation but not induce apoptosis. However, RAD001 in combination with cisplatin can induce a remarkable increase in the number of apoptotic cells both in vitro and in vivo by downregulating the expression of prosurvival molecules such as Bcl-2 and survivin [79]. Similarly, the combination of rapamycin with doxorubicin displayed better anticancer effect compared to either of the single agent treatments [83]. Moreover, mTOR inhibitor attenuated the doxorubicin-induced inhibition of endothelial cell proliferation. For example, doxorubicin can stimulate expression of p21 which was reversed by the addition of rapamycin. Furthermore, mTOR can inhibit HCC development in synergy with many other anticancer agents such as 5-fluorouracil (5-Fu) [81], microtubule inhibitors [84], and vinblastine [82]. In addition to chemotherapy drugs, target therapy agents such as proteasome inhibitor, bortezomib, can synergize with rapamycin to reduce growth, repress mobility, and induce apoptosis of HCC cells [85]. Another phase 1 trial of temsirolimus combined with sorafenib showed that the maximum-tolerated dose (MTD) was temsirolimus 10 mg weekly plus sorafenib 200 mg twice daily [74]. Grade 3 or 4 adverse events were hypophosphatemia, infection, thrombocytopenia, hand-foot skin reaction (HFSR), and fatigue. Two of 25 patients had a PR and 15 of 25 had SD. There is another trial that showed that the MTD of everolimus in combination with standard dose sorafenib was 2.5 mg daily [75]. Median time to progression was 4.5 months and overall survival was 7.4 months. Most common adverse events are diarrhea, HFSR, and thrombocytopenia. In addition to chemotherapy and target therapy, the effect of radiation therapy can also be augmented by mTOR inhibitors. For example, RAD001, at clinically relevant nanomolar concentrations, enhanced the efficacy of radiation in HCC cells. The induction of autophagy may account for this effect [86].

Although mTOR inhibitors have shown great potential for the treatment of HCC patients, surrogate biomarkers are necessary to identify suitable patients so as to improve clinical efficacy and prevent drug resistance. For example, rapamycin can activate PI3K-Akt in an insulin-like growth factor-dependent manner by relieving S6K1-dependent inhibitory phosphorylation of IRS-1, thus preventing IRS-1 degradation and enhancing PI3K activation. Rapamycin can inhibit S6K1-dependent IRS-1 serine phosphorylation, increase IRS-1 protein levels, and promote association of tyrosine-phosphorylated IRS-1 with PI3K [87]. Such a negative feedback regulation is important to maintain homeostasis. However, the disruption of this negative feedback by mTOR inhibitors may attenuate the therapeutic effect. Fortunately, there are also some agents that have multiple targets including mTOR and PI3K. For example, NVP-BEZ235 is a dual inhibitor of PI3K and mTOR. It can decrease the levels of p-Akt and p-S6K and repress cell proliferation in HCC cell lines [88]. Moreover, it can repress tumor growth without loss of body weight. Interestingly, combination of everolimus with NVP-BEZ235 can synergistically suppress the proliferation of HCC cells [89]. PI-103, a dual PI3K/mTOR inhibitor, in combination with sorafenib can effectively inhibit the proliferation of HCC cells by blocking both Ras/Raf/MAPK and PI3K/Akt/mTOR pathways [90].

## 6. Conclusions and Perspectives

mTOR plays an important role in viral hepatitis and HCC development. mTOR inhibitors can repress cell growth both in vitro and in vivo. Preliminary clinical trials indicated that mTOR inhibitors alone or in combination with cytotoxic drugs or targeted therapy drugs can improve clinical outcomes of HCC patients. However, no RCTs have proven the benefits of everolimus treatment in HCC. Novel biomarkers are warranted to identify suitable HCC patients who may benefit from the treatment of mTOR inhibitors. Although the most common adverse events are tolerated, it is noteworthy that mTOR is associated with HBV virus replication and mTOR inhibitors may cause hepatitis B reactivation. HCC cells are prone to develop multiple drug resistance due to the heterogeneity and fragile genome. Therefore, the combination of mTOR inhibitors with conventional chemotherapy drugs and target therapy agents might be a promising strategy for the future application of mTOR inhibitors.
